# Electronic Cigarette Smoke Impairs Normal Mesenchymal Stem Cell Differentiation

**DOI:** 10.1038/s41598-017-14634-z

**Published:** 2017-10-27

**Authors:** A. Shaito, J. Saliba, A. Husari, M. El-Harakeh, H. Chhouri, Y. Hashem, A. Shihadeh, M. El-Sabban

**Affiliations:** 1 Department of Biological and Chemical Sciences, Faculty of Arts and Sciences, Lebanese International University, Beirut, Lebanon; 2 Department of Biology, Faculty of Science, Lebanese University, Beirut, Lebanon; 3 Department of Internal Medicine, Faculty of Medicine, American University of Beirut, Beirut, Lebanon; 4 Department of Anatomy, Cell Biology and Physiological Sciences, Faculty of Medicine, American University of Beirut, Beirut, Lebanon; 5 Department of Mechanical Engineering, Faculty of Engineering, American University of Beirut, Beirut, Lebanon

## Abstract

Electronic cigarettes (e-cigarettes) are promoted as low-risk alternatives to combustible cigarettes. However, the effects of chronic inhalation of potential toxicants emitted by ecigarettes remain largely unexamined. It is conceivable that smoking-induced chronic diseases result in cellular injury, in the absence of effective repair by stem cells. This study evaluates the effect of cigarette and e-cigarette aerosol extracts on the survival and differentiation of bone marrow-derived mesenchymal stem cells (MSCs). MSC growth and osteogenic differentiation were examined after exposure to smoke extracts. Data revealed detrimental effects of both cigarette and e-cigarette extracts on MSC morphology and growth. Levels and activity of alkaline phosphatase, an osteogenic marker, decreased and induction of osteoblastic differentiation was impaired. Both smoke extracts prevented osteogenic differentiation from progressing, evident by decreased expression of terminal osteogenic markers and mineralization. Elevated levels of reactive oxygen species (ROS) were detected in cells exposed to smoke extracts. Moreover, decreased differentiation potential was concomitant with severe down-regulation of Connexin 43 expression, leading to the loss of gap junction-mediated communication, which together with elevated ROS levels, could explain decreased proliferation and loss of differentiation potential. Hence, e-cigarettes present similar risk as combustible cigarettes with respect to tissue repair impairment.

## Introduction

The detrimental impact of cigarette smoking on health is amply documented and ranges from oral diseases^1^, to systemic malfunction, inflammation^2^, infertility^3,4^, cancer and abnormal cell differentiation and tissue repair^1^. Awareness has been raised among smokers and policy-makers, and has resulted in proactive measures aiming at curbing cigarette smoking. Controversially, waterpipe smoking is gaining popularity worldwide, alongside another globally spreading phenomenon, the use of electronic cigarette (e-cigarette) or “vaping”^5^.

E-cigarettes are often claimed to be a safer alternative to conventional tobacco products and are sometimes marketed as a smoking cessation tool. Some research has suggested a decrease in the disease burden of e-cigarette vaping, compared to combustible cigarette smoking^6^. However, e-cigarette liquids have been reported to be cytotoxic^7,8^, and e-cigarette aerosol emissions have been shown to exert negative effects in animal models^9–14^. Nevertheless, partly due to the recent emergence of the e-cigarette, there is a lack of information on its long-term effects on health and studies on e-cigarette safety are not yet conclusive. Combustible cigarette smoke compromises cell growth and tissue repair^1,15,16^; however, the impact of e-cigarette aerosols on cell differentiation and tissue repair has not been studied.

A stable epithelial layer with a relatively slow cell turnover rate lines the respiratory tract^17^. Upon injury, progenitor and stem cells are recruited to repair damaged tissues. However, smokers develop chronic conditions, from long-term exposure to smoke, suggesting impaired tissue healing and remodelling. Previously, we explored the effect of waterpipe smoke on alveolar type II-derived cells^18^ and on endothelial cells^19^, detailing cytotoxic, mutagenic, inflammatory and anti-proliferative effects. The onset of systemic inflammation and the compromised ability of local cells to heal the damaged tissues were proposed as a plausible mechanism underlying tobacco smoke-induced diseases such as chronic obstructive pulmonary disease (COPD) and vascular diseases^18,19^. These conditions remain with no cure and a rather modest clinical management^20,21^.

Stem cells are at the core of tissue repair and remodelling. Bone marrow-derived mesenchymal stem cells (MSCs) are frequently recruited to the site of injury^22^ and are extensively studied for the treatment and repair of tissues such as in cardiac injury^23,24^. Among the documented hazards associated with smoking, generation of reactive oxygen species (ROS) and alteration of gap junctional complexes are tightly associated with modulation of repair mechanisms. Indeed, multiple studies have highlighted the importance of gap junctions in protecting cells against oxidative stress-induced cell death^25^ and in modulation of cell proliferation and survival^25,26^, tumorigenesis^27^, and differentiation^28,29^. Nicotine was shown to down-regulate the expression levels of Connexin 43 (Cx43) in human endothelial cells^30,31^, which affects viability, proliferation, and angiogenesis^32^. In addition, low Cx43 expression is strongly associated with the metastatic phenotype of cancer cells^33,34^, while up-regulation of Cx43 expression restores the sensitivity of lung carcinoma cells to chemotherapy *in vitro* ^35^. Cx43 is reported to be highly expressed in MSCs^36^. MSCs undergoing osteoblastic differentiation express Cx43; Cx43 plays a major role in osteoblast progenitor migration and homing to bone *in vivo* ^28,37–39^.

The present study investigates the effect of combustible cigarette and e-cigarette smoke extracts on the ability of stem cells to differentiate, as impairment of stem cell differentiation leads to tissue repair impairment, causing chronic diseases.

## Results

### Smoke extracts inhibit MSC proliferation and induces morphological changes

**Figure 1:**
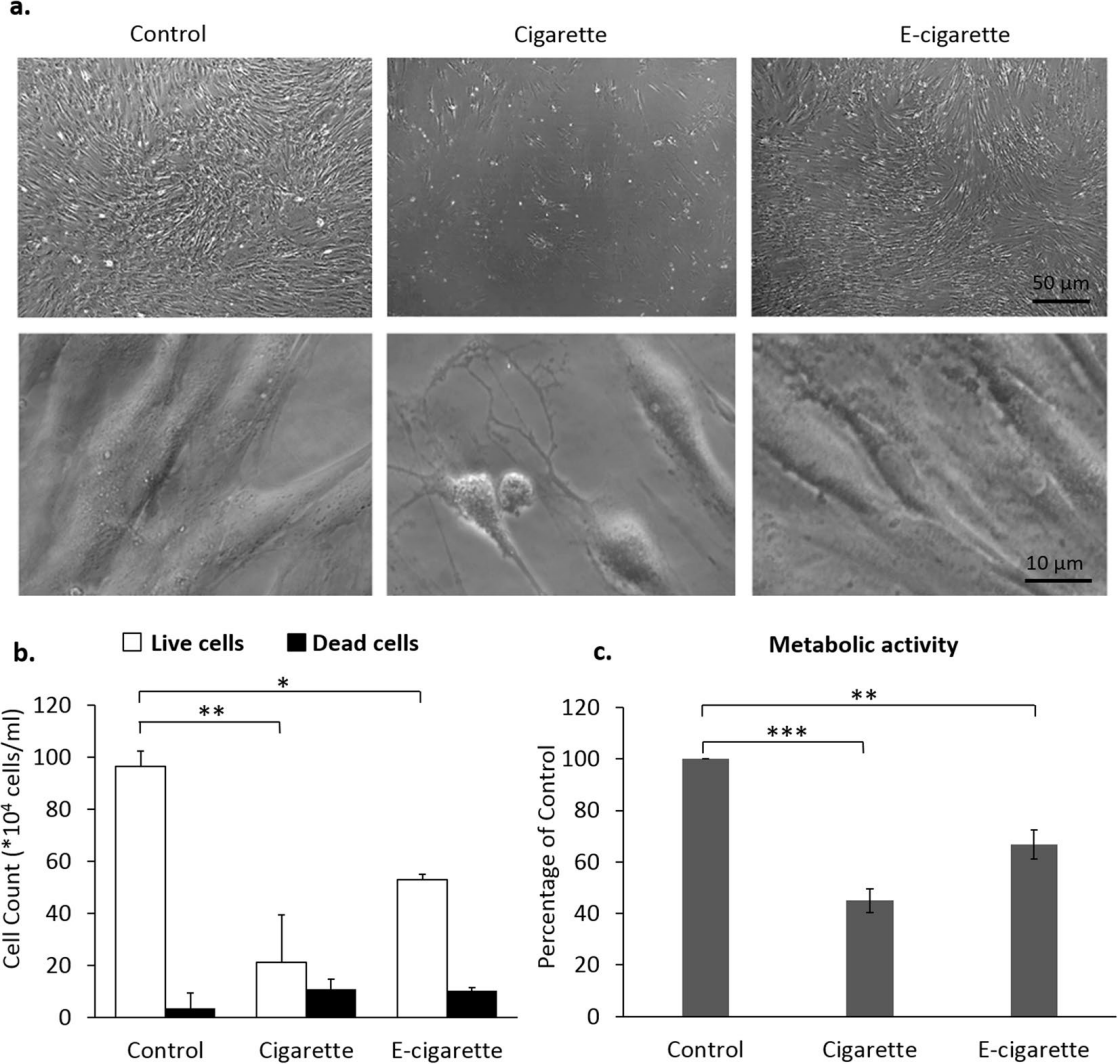
Cigarette and e-cigarette smoke extracts alter MSC morphology and growth. MSCs were repeatedly (once every 3 days) exposed to cigarette and e-cigarette smoke extracts for 21 days. (**a**) MSC morphology was examined by light microscopy (upper panel). High magnification DIC images are shown in the lower panel. (**b**) On day 21, cell viability was assessed using the trypan blue dye exclusion assay. Histograms display averages ± SD of 3 independent experiments in duplicates. (**c**) On day 21, the metabolic activity of MSCs was assessed by MTT. Histograms display averages ± SD of 2 independent experiments in triplicates. *p < 0.01, **p < 0.005 and ***p < 0.001.

The concentration of smoke extract to be used throughout the study was determined by exposing MSCs to increasing concentrations of smoke extracts. Media were replenished every third day over 12 days. Treated MSCs showed poor viability at concentrations higher than 0.6 mg/ml and 6 mg/ml for cigarette and e-cigarette extracts, respectively. At higher concentrations, cells stopped proliferating, acquired spindle-like morphology with long processes and, eventually, sloughed off. Therefore, for the aims of this study, the concentrations of 0.4 mg/ml and 4 mg/ml were chosen for the cigarette and e-cigarette sets, respectively, applied every third day, for 3 weeks. We note that the tenfold difference in concentration between e-cigarette and cigarette extracts is consistent with the approximately tenfold greater amount of aerosol particulate matter inhaled by an e-cigarette user for the same nicotine delivery. By day 21 of the experiment, in response to repeated exposure, cells underwent morphological alterations that were more pronounced in the cigarette- than in e-cigarette samples (Fig. 1a). At high magnification, microscopic images of MSCs treated with smoke extracts display altered morphology with variable response to the insult ranging from mild changes to severe shrinkage and collapsing of cytoplasm. Morphological alteration were observed, albeit to a lesser extent, in cells treated with e-cigarette smoke extracts with overall smaller cells, compared to control MSCs (Fig. 1a; lower panel). Trypan blue exclusion assay showed an 80% (*p* < 0.005) and 50% (*p* < 0.01) decrease in the number of cigarette- and e-cigarette-treated cells, respectively (Fig. 1b). The extent of cell death was also measured in cigarette- and e-cigarette-treated wells, where the number of dead cells was threefold higher in both groups, as compared to control. This trend in cell viability was paralleled and confirmed by a comparable decrease in the metabolic activity of the cells. Indeed, the MTT assay showed a 50% (*p* < 0.001) and 30% (*p* < 0.005) decrease in the metabolic activity of cigarette- and e-cigarette-treated cells, respectively (Fig. 1c). These data indicate that both cigarette and e-cigarette particles compromise MSCs proliferation.

### Smoke extracts impair MSCs differentiation

**Figure 2:**
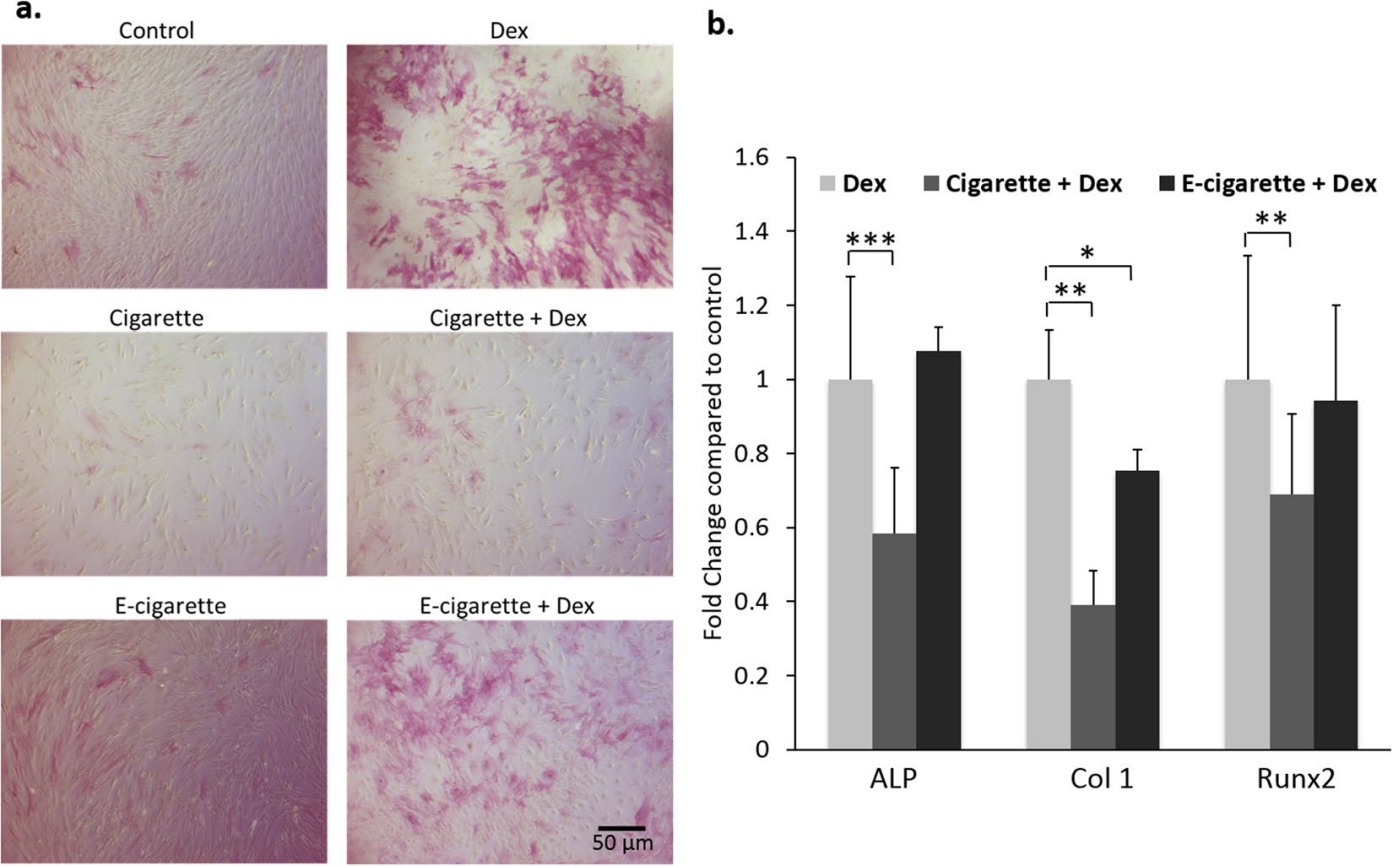
Cigarette and e-cigarette smoke extracts attenuate MSC differentiation. (**a**) MSCs were induced into osteogenic differentiation by treatment with 50 nM Dex for 14 days. ALP activity was then assessed using a chromogenic ALP assay where purple staining indicates ALP activity. The light microscopy images are representative of 3 independent experiments. (**b**) MSCs were simultaneously treated with 50 nM Dex for osteogenic differentiation and cigarette or e-cigarette smoke extracts for 14 days. Expression of osteogenic markers (alkaline phosphatase [ALP], collagen 1 [Col 1] and Runt-related transcription factor 2 [Runx2]) was then assessed using qRT-PCR. Histograms display data normalized against GAPDH and show fold changes relative to control. Results are displayed as averages ± SD of 3 independent experiments. *p < 0.05, **p < 0.01 and ***p < 0.001.

The potential of MSCs to differentiate was evaluated by inducing osteoblastic differentiation using 50 nM Dex. The effect of repeated exposure to smoke extract on the induction of MSCs differentiation, as monitored by the expression of different osteogenic differentiation markers, ALP, Col 1, and Runx2 was assessed. Dex induced ALP activity (Fig. 2a) and ALP gene expression in MSCs (Fig. 2b). Both cigarette and e-cigarette reduced basal ALP activity (Fig. 2a). Only cigarette smoke extracts significantly decreased ALP mRNA levels upon treatment with Dex (*p* < 0.0001) (Fig. 2b). Given the role of ALP as an early osteogenic marker and that Dex drives MSCs to early osteogenic differentiation, these data indicate that early osteogenic differentiation is less affected by e-cigarette than by cigarette smoke extracts. In addition, the expression of Col 1 and Runx2 decreased in smoke extracts and Dex-treated MSCs (*p* < 0.01) (Fig. 2b). Only Col 1 mRNA levels decreased significantly upon e-cigarette smoke extract treatment (*p* < 0.05).

**Figure 3:**
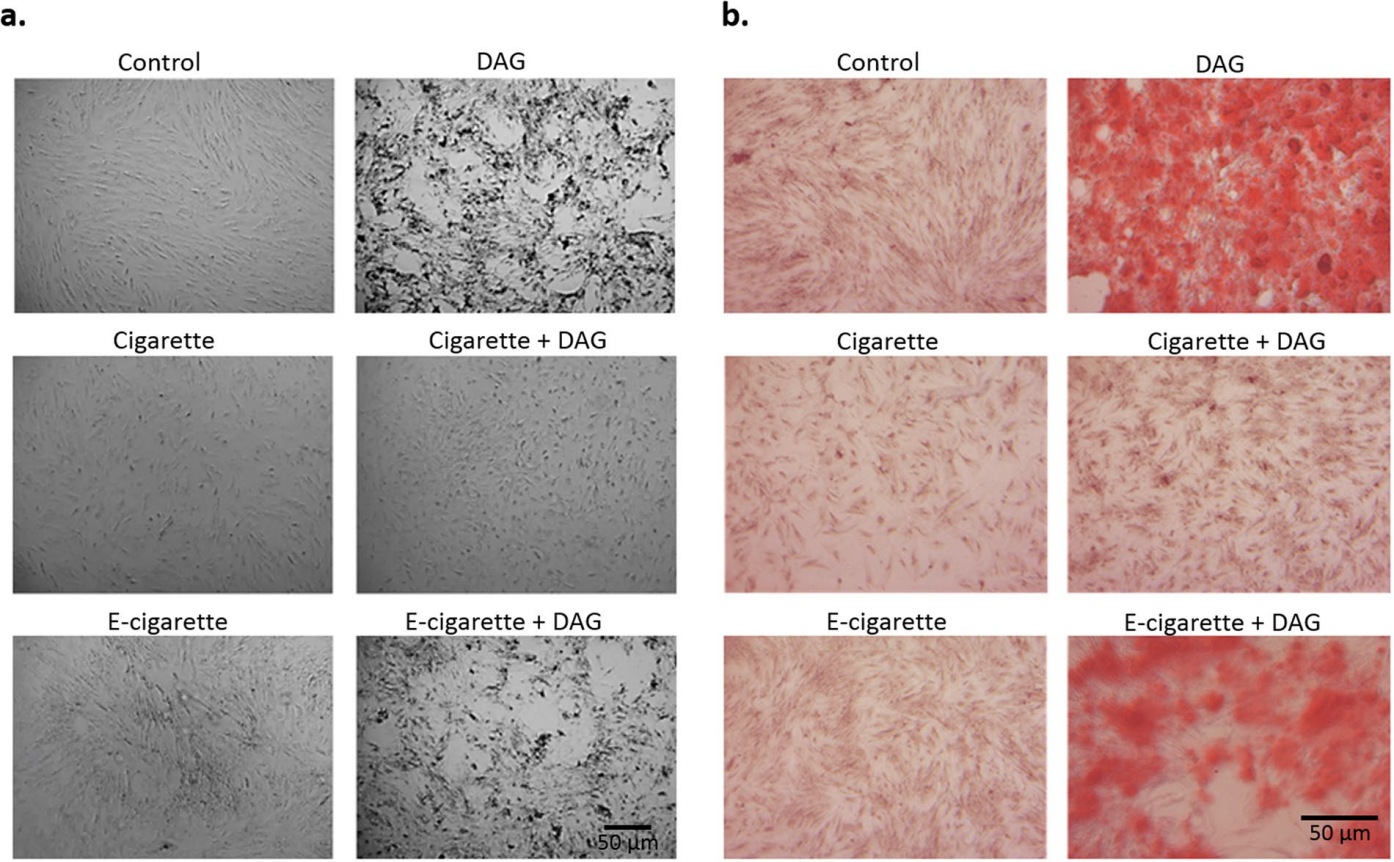
Cigarette and e-cigarette smoke extracts block progress towards terminal osteogenic differentiation. MSCs were treated with cigarette and e-cigarette smoke extracts in the presence or absence of DAG for 21 days. (**a**) Cell morphology was observed under the light microscope. Mineral deposition in the plates is evident as black spots. (**b**) MSCs were stained with Alizarin Red S stain and observed under the light microscope. Orange staining indicates mineralized calcium deposits. Images are representative of 3 independent experiments.

DAG treatment drives MSCs beyond the early differentiation induced by Dex alone. DAG induces extensive morphology change in MSCs, which became rounded with deposits visible in the background; however, smoke impeded DAG-induced morphological changes (Fig. 3a). Alizarin Red S staining confirmed osteogenic differentiation in DAG-treated cells that showed dense red-orange patches, attesting to mineralization (Fig. 3b). A much less pronounced Alizarin Red S staining was observed in smoke-treated MSCs. This suggests that not only cigarette, but also e-cigarette smoke extracts largely impair the progress of differentiation beyond ALP expression.

### E-cigarette smoke extracts lead to overproduction of ROS

**Figure 4:**
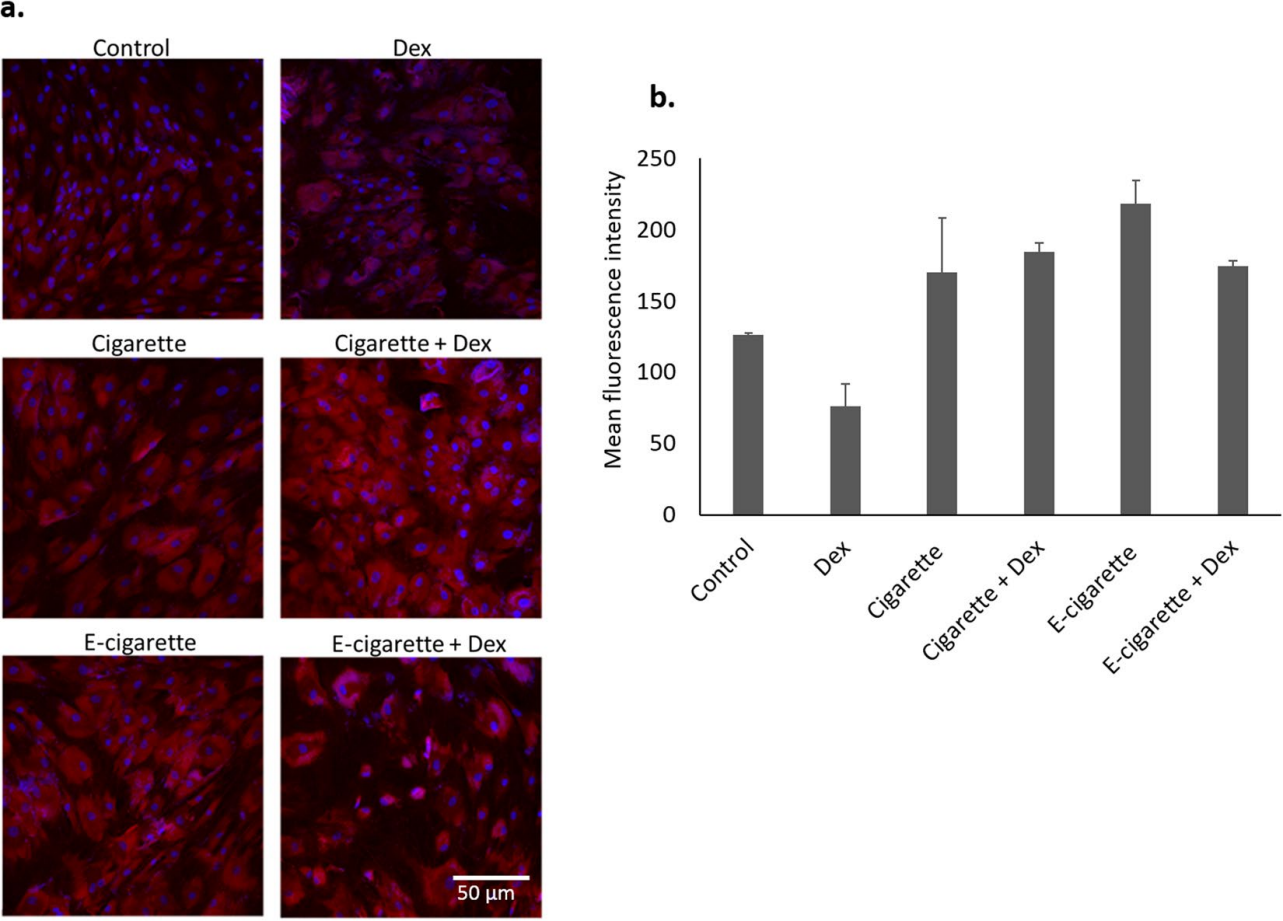
Cigarette and e-cigarette smoke extracts enhance ROS production. (**a**) MSCs were treated with cigarette and e-cigarette smoke extracts for 14 days. ROS generation was evaluated using the DHE assay, counterstained with DAPI and observed by fluorescence microscopy. Red indicates presence of ROS. (**b**) Quantification of ROS generation. DHE mean fluorescence intensities of 5 fields from 2 images per condition were measured using the Zen 2011 software. Histograms display averages ± SD of mean fluorescence intensities.

The impaired differentiation of MSCs upon cigarette and e-cigarette challenge could be due to several factors. ROS are important upstream osteogenic differentiation mediators in MSCs. ROS levels were visualized in control and smoke-exposed MSCs to determine whether e-cigarette extracts induce ROS generation and whether high levels of ROS may be involved in hindering osteogenesis. Figure 4a shows that MSCs exhibit high levels of ROS in response to smoke extract, as compared to control. Control cells had a mean fluorescence intensity (MFI) of 126 ± 1.4, while cigarette-treated cells had MFI of 170 ± 38.2 (Fig. 4b) and ROS levels were highest in e-cigarette-treated MSCs (MFI = 218 ± 16.2). Furthermore, differentiated cells exhibited low levels of ROS (Fig. 4a, left lower panel), with MFI = 76 ± 15.5, while smoke-exposed cells also treated with Dex maintained elevated ROS levels, which could, in part, explain smoke-induced inhibition of osteogenic differentiation.

### Cigarette and e-cigarette smoke extracts inhibit cell-cell communication

**Figure 5:**
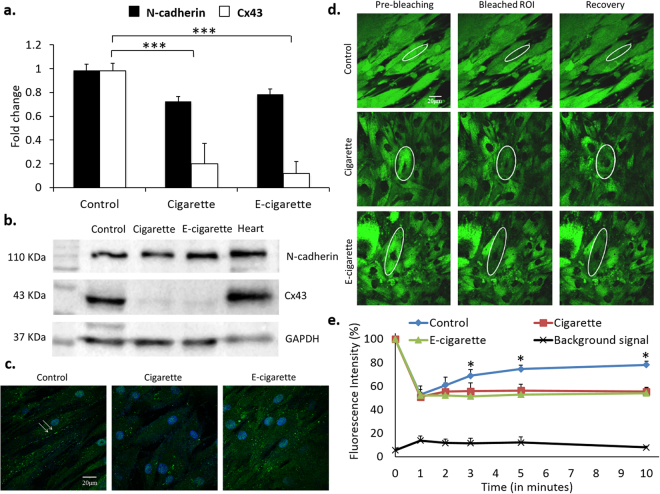
Cigarette and e-cigarette smoke extracts inhibit cell-cell communication in MSCs. MSCs were treated with cigarette and e-cigarette smoke extracts for 14 days. (**a**) qRT-PCR of Cx43 and N-cadherin was performed on total RNA obtained from control and treated cells. Data were normalized against GAPDH and show fold changes (averages ± SEM of 3 independent experiments) relative to the control. (**b**) Western blots of Cx43 and N-cadherin were performed on total protein extracts obtained from control and treated cells. GAPDH was used as a loading control and total heart lysates as a technical positive control. Blots are representative of 3 independent experiments. (**c**) Immunofluorescence staining of Cx43 was performed on control and treated MSCs. Arrows indicate gap junction plaques between adjacent cells. Images are representative of at least 10 fields from 2 independent experiments. (**d**) MSCs from the control and treated wells were stained with calcein-AM and then subjected to 50% photo-bleaching by the 488-nm laser. Fluorescence recovery after photo-bleaching (FRAP) was monitored for 10 minutes under the 63X objective. White ellipses indicate regions of interest (ROI). Images are representative of at least 5 ROIs per condition, from 2 independent experiments. (**e**) Quantification of fluorescence intensity of ROIs relative to reference cells of control and treated cells. Values represent the fluorescence intensity (averages ± SD) of each ROI based on several measurements calculated by the Zeiss Zen 2011 software. *p < 0.05 and ***p < 0.001.

In order to further explore the mechanisms behind MSCs growth and differentiation impairment, the integrity of cell-cell communication was assessed upon exposure to smoke extracts. Figure 5a shows a marked decrease in Cx43 mRNA levels. Western blot and immunofluorescence (Fig. 5b,c) show a sharp downregulation of Cx43 protein levels in both cigarette- and e-cigarette-treated cells. N-cadherin, a component of junctional complexes involved in cell-cell contact, was also examined and its expression was maintained both at the mRNA and protein levels (Fig. 5a,b). This suggests that the integrity of the cell layer is preserved and that downregulation of Cx43 is a specific effect of smoke exposure and not secondary to the disruption of cell membrane integrity and cell-cell contact. Downregulation of Cx43 was further investigated at the functional level. FRAP studies show that smoke-treated cells do not recover fluorescence after photo-bleaching to at least 50% of their initial fluorescence intensity (Fig. 5d,e). The background fluorescence intensity is maintained throughout the experimental duration (black curve, Fig. 5e). Cigarette- and e-cigarette-challenged cells did not recover fluorescence, compared to a 30-40% recovery in control cells (blue curve). Indeed, fluorescence recovery between minute 1 and minute 10 was not significant in the cigarette (*p* = 0.32) or in the e-cigarette (*p* = 0.52) treated cells, while in the untreated cells, fluorescence recovery was significant at 10 minutes (*p* < 0.005). Similarly, at the end of recovery period (10 minutes), fluorescence difference between treated and control cells was significant (*p* < 0.05), while the difference of fluorescence intensity between cigarette and e-cigarette treated cells was not significant (*p* = 0.25). Both cigarette and e-cigarette smoke extracts compromised gap junction-mediated intercellular communication.

## Discussion

Currently, there is no consensus on the use of e-cigarettes as nicotine surrogates or as smoking cessation tools and the concept remains controversial due to the relatively recent emergence of “vaping” trends^40^ and a paucity of data on its net health effects in the population. Understandably, much attention to date has focused on e-cigarette aerosol toxicant content^41–51^, and particularly those toxicants thought to be causative agents in cigarette smoke-related disease – the so-called Hoffmann analytes^52^. E-cigarette proponents have pointed out that under most conditions, e-cigarettes emit fewer and less Hoffmann analytes than do combustible cigarettes. While toxicant analysis is a powerful tool for studying the potential risk of product exposure, it is not generally known *a priori* which toxicants to scan; indeed, due to their different sources, e-cigarette aerosol toxicity may be due to constituents not included in the Hoffmann list. Recent literature on the effects of e-cigarette on animals and cells^10–14,50,53,54^ suggests that the relatively low amounts of Hoffmann analytes in e-cigarette aerosols may not provide an adequate picture of the possible effects of long-term use. This study is one of the earliest works that examined the potential effects of e-cigarette aerosol extracts on human stem cells, suggesting that e-cigarette smoke particles may adversely impact human health.

The role of stem cells and their capability to differentiate and repair organs damaged by smoking is crucial in diseases associated with tobacco use like COPD. This study compared the effect of exposure to combustible cigarette and e-cigarette smoke extracts on the survival of stem cells and their differentiation potential. As a proof of concept, and due to the relative ease of induction and assessment of differentiation in MSCs, the difficulty in obtaining organ-specific stem cells, and the fact that MSCs are recruited to sites of injury for tissue repair, the well-established model of MSC differentiation into osteoblast-like cells was used to assess the effects of smoke extracts on the differentiation potential of MSCs. Our results demonstrated that both cigarette and e-cigarette smoke extracts significantly affect the proliferation of MSCs. This finding was in agreement with a previous study that demonstrated the attenuation of A549 alveolar cellular growth secondary to cigarette exposure^9^. This loss of proliferative potential was accompanied by morphological changes, also indicative of alterations in cell behaviour. Addition of Dex to induce MSCs into osteoblastic differentiation did not salvage the cells and failed to induce MSC differentiation in the cigarette-treated samples. E-cigarette-treated MSCs, however, seemed to retain differentiation potential in the presence of Dex, though to a lesser extent than control cells. However, attempts to progress MSC differentiation by the addition of DAG failed in the treated cells. Exposure to either cigarette or e-cigarette smoke extracts compromised response of MSCs to DAG, as evidenced by the absence of differentiation features (mineralization and calcium deposition).

MSC differentiation potential is modulated by the type and level of ROS^55–57^; indeed, MSC osteogenesis occurs under low ROS levels^58–60^. Cigarette smoke contains high concentration of ROS-inducing agents^61^, which are known to decrease the differentiation potential of MSCs into osteoblasts^62^. MSCs exposed to both cigarette and e-cigarette smoke extracts generated high levels of ROS, as defined by increased superoxide radical levels^54,63,64^, which can explain attenuated differentiation of smoke-exposed cells^65^.

Gap junction-mediated intercellular communication is pivotal to the regulation of several cellular processes including proliferation, differentiation, apoptosis and tumorigenesis^66^. Cx43 is the main connexin in bone marrow-derived MSCs^36^ and appears to be directly involved in osteogenic differentiation. Our data suggest that the loss of Cx43, upon exposure to cigarette and e-cigarette smoke extracts, and the subsequent loss of cell-cell communication, is associated with the decrease in the differentiation potential and hence the attenuated potential of MSCs to undergo osteogenic differentiation. Cx43 also protects cells from oxidative stress^25^, and the down-regulation of Cx43 can explain impaired growth and differentiation of MSCs upon exposure to the combination of smoke and Dex. This observation may suggest that e-cigarette can be a risk factor for osteoporosis, a chronic condition tightly linked to cigarette smoking^67^ and comorbidity with COPD^68^. Our results suggest that smoke extracts might be inhibiting the cytoplasmic exchange of survival and differentiation signals, by down-regulating Cx43 expression in MSCs. In addition to the fact that nicotine might be a player in the impairment of differentiation^69^, smoke contains other ingredients that are toxic and could impair differentiation as well^70,71^. Subsequently, tissue repair might be compromised upon exposure to both cigarette and e-cigarette.

Therefore, while e-cigarette aerosols are commonly understood to contain fewer and less of the toxicants that have been linked to diseases in combustible cigarette smokers, this study adds to the growing evidence that suggests that a cautionary approach to e-cigarette proliferation is necessary.

## Material and Methods

### Smoke extract preparation

Combustible and electronic cigarette aerosols were generated using the Oro-nasal Respiratory Exposure System (ONARES, CH Technologies, USA), as previously described^9^. Briefly, combustible cigarette particulate matter was generated from reference 3R4F cigarettes (University of Kentucky, Lexington, KY) with 9.4 mg tar, and 0.726 mg nicotine per cigarette, following a puffing protocol of one 2-second puff every minute and a volume of 35 ml/puff (ISO standard). E-cigarette particulate matter was obtained from pre-filled V4L CoolCart (strawberry flavor, 3.5 Ohm, 18 mg/ml labelled nicotine concentration) cartomizer cartridges; by 4-second puff duration, 1.2 l/minute flow rate, and 14-second inter-puff interval^72^.

Aerosol particles were trapped onto 47-mm glass fiber filter disks type A/E (Pall Laboratory, USA) and then extracted in cell culture medium using a syringe and sterilized through a 0.22-μm filter (Costar, Corning Inc., New York, USA), to the final concentrations to be applied onto cells.

### Cells and cell culture

Bone marrow-derived mesenchymal stem cells (MSCs), isolated from healthy volunteers, were purchased from Lonza (Cologne, Germany). These MSCs retain the ability to self-renew and differentiate into multiple mesodermal lineages (osteogenic, adipogenic, chondrogenic and bone marrow stroma). MSCs were cultured in complete Dulbecco’s Modified Eagle’s Medium (DMEM), supplemented with glucose (1 g/l), fetal bovine serum (10%) and penicillin/streptomycin (100 units of potassium penicillin and 100 µg of streptomycin sulphate per 1 ml of DMEM).

Smoke extracts were freshly prepared prior to addition to cells. Ascending concentrations of combustible and e-cigarette total smoke extracts were applied onto the cells for 2 weeks. Cells were monitored daily by microscopy to determine the concentration to be used to enable cellular and molecular observations.

Dexamethasone (Dex) (DEXAMED, Medochemie, Limassol, Cyprus) was used at a concentration of 50 nM to induce osteogenic differentiation^73,74^, added onto the cells either alone or simultaneously with the smoke extract. To further differentiate MSCs into osteoblast-like cells, an osteogenic differentiation combination was used (50 nM Dex, 50 μM ascorbic acid and 10 mM beta-glycerophosphate, abbreviated as DAG).

MSCs were plated at a density of 7,000 cells/cm^2^. Smoke extract exposure and differentiation started when cells reached 70% confluence; treatment was applied every third day, for a total of 14 or 21 days.

### Morphological and histochemical assessment

MSCs were seeded onto 6-well plates (Corning, New York, USA) and treated with smoke extracts +/− Dex (14 days) or +/−DAG (21 days).

### Morphological changes

Cells were monitored daily by light microscopy and images were captured to document changes in cell confluence and morphology.

### Cell viability

Cell growth was assessed using the trypan blue exclusion assay. Control and treated cells were plated in duplicates in 24-well plates. At pre-determined time points, MSCs were released from the plate using 0.05% Trypsin-EDTA (R-001-100, Life Technologies, California, USA), then equal volumes of cells and trypan blue dye and counted using a haemocytometer.

In parallel, the MTT assay was performed to evaluate the metabolic activity of MSCs. Control and treated cells were seeded in duplicates onto flat-bottom 96-well plates. At each time point, cells in 90 µl media were incubated with 10 µl of a 5 mg/ml MTT substrate solution, thiazolyl blue tetrazolium bromide salt (Sigma-Aldrich Co, Missouri, USA) for 4 hours. The reaction was stopped by the addition of 100 µl of solubilizing solution (12 mM HCl, 346 mM SDS and 5% isobutanol); formazan dye was then quantified at a wavelength of 595 nm using a scanning multi-well spectrophotometer (Thermo Scientific Multiskan EX, Thermo Scientific, USA). Proliferation results were reported as percentages of control.

### Alkaline phosphatase activity

Alkaline phosphatase (ALP) is an enzyme highly active in bone tissue. ALP activity was semi-quantitatively evaluated using the Leukocyte Alkaline Phosphatase Kit (R86, Sigma-Aldrich, Missouri, USA). On day 14, cells were washed in phosphate-buffered saline (PBS), fixed and stained for ALP activity, according to the manufacturer’s instructions. Purple dye deposits indicate cells with ALP activity.

### Alizarin red staining

On day 21, MSCs were washed in PBS, fixed in a solution of equal volumes of acetone and xylene for 30 minutes, and rinsed twice in distilled water. Cells were stained with Alizarin Red S (40 mM) for 20 minutes at room temperature, with gentle rocking then washed in distilled water to remove excess dye and left to air dry. Dark orange-red patches indicate areas of osteogenic mineralization. Images were captured using a Zeiss Axiovert microscope (Primovert HDcam, Carl Zeiss, Germany).

### Reactive oxygen species generation

MSCs were treated with cigarette and e-cigarette extracts +/− Dex for 14 days (4 repeated applications of the experimental media). Cells were washed in PBS and incubated for 30 minutes at 37 °C with 10 μM dihydroethidium (DHE). DHE reacts with ROS, to produce red fluorescent 2-hydroxyethidium^63^. Cells were counterstained with DAPI. Images were captured and analyzed using a laser-scanning confocal microscope (LSM 710, Carl Zeiss, Germany), operated by the Zen 2011 software. DHE mean fluorescence intensities of 5 fields from 2 separate images per condition (obtained using fixed acquisition settings for comparison purposes) were measured using the Zen 2011 software. Histograms display averages ± standard deviations (SD) of mean fluorescence intensities.

### Molecular analysis

#### Gene expression by quantitative PCR

Total RNA was extracted from smoke extract-treated and control MSCs 21 days after culture using the NucleoSpin^®^ RNA II extraction kit (Macherey-Nagel, Düren, Germany) as per manufacturer’s instructions. Total extracted RNA was then reverse-transcribed with the RevertAid^®^ first strand cDNA synthesis kit (Thermo Scientific), using random primers, following the manufacturer’s protocol. cDNA was amplified using an iQ SYBR Green Supermix (BioRad Laboratories, Hercules, California, USA) on a BioRad CFX96 real-time PCR system. Cycling conditions were as follows: 95 °C for 3 minutes followed by 40 amplification cycles (95 °C denaturation for 3 seconds, annealing for 30 seconds at the primers’ melting temperature, and extension at 72 °C for 30 seconds) and a final extension cycle at 72 °C for 5 minutes. Relative expression of target genes was performed according to the comparative ∆∆Ct method using human glyceraldehyde 3-phosphate dehydrogenase (gapdh) as a reference gene for normalization. Gene expression levels were assessed for ALP, Collagen 1A1 (Col 1) and runt-related transcription factor 2 (Runx2). The primers used were as follows: ALP 5′-acaagcactcccacttcatctgga-3′ and 5′-tcacgttgttcctgttcagctcgt-3′; Col 1 5′-ttttgtattcaatcactgtcttgcc-3′ and 5′-cagccgcttcacctacagc-3′ and Runx2 5′-tccggaatgcctctgctgttatga-3′ and 5′-aaggtgaaactcttgcctcgtcca-3′.

#### Protein expression by western blot

Total cell lysates were obtained by scraping cells in 2 µl/cm^2^ of sample buffer (126 mM Tris/HCl, 20% glycerol (v/v), 40 mg/ml of sodium dodecyl sulphate [SDS]). Collected cell lysates were quantified using the BioRad DC quantification kit according to the manufacturer’s instructions. After heating at 95 °C for 5 minutes and addition of 0.7 M β-mercaptoethanol and 5 µl of bromophenol blue, 50 µg of proteins were loaded on a 10% SDS-PAGE gel and transferred onto a methanol-activated PVDF membrane. Membranes were blocked in fat-free milk (5% w/v) and probed with the following antibodies: mouse monoclonal antibody against human N-cadherin (33–3900, Invitrogen, California, USA); Rabbit polyclonal antibody against human Cx43 (71-0700, Life Technologies, California, USA). Membranes were then incubated with the appropriate horseradish peroxidase (HRP)-conjugated secondary antibody and proteins were detected by chemoluminescence (Santa Cruz technologies, California, USA). Equal loading was assessed using mouse monoclonal HRP-conjugated antibody against the housekeeping enzyme GAPDH (Abnova Corporation, Taipei, Taiwan). Images were captured using the BioRad Chemidoc MP system.

#### Protein localization by immunohistochemistry

MSCs were seeded onto sterile coverslips in 24-well plates and treated with cigarette or e-cigarette extracts for 14 days. At the end of the experimental duration, cells were washed in PBS, fixed and permeabilized in ice-cold absolute ethanol. Cells were blocked with 3% (v/v) non-immune goat serum (NGS) in PBS for one hour at room temperature. Cx43 antibody, diluted in 1% (v/v) NGS in PBS to the concentration of 1 μg/ml, was added onto the cells, overnight at 4 °C in a humidified box. Labelling was achieved by the addition of Alexa 488-labelled fluorescent goat-anti-rabbit secondary antibody. Cell nuclei were stained with 1 μg/ml Hoechst 33324 solution (H3570, Molecular Probes, New York, USA). Finally, ProLong Antifade reagent (P36930, Molecular Probes, New York, USA) was used to cover the cells. Images were acquired using the LSM 710.

### Intercellular communication by fluorescence recovery after photo-bleaching (FRAP)

Functionality of gap junction-mediated intercellular communication upon smoke exposure was assessed using the FRAP assay. MSCs were seeded onto confocal dishes (MatTek Corporation, Massachusetts, USA) and treated with cigarette or e-cigarette extracts. On day 14, cells were labelled with calcein-AM (C3100, Molecular Probes, New York, USA), by incubation in 1 μM calcein-AM reconstituted in DMSO, for one hour at 37 °C. Experiments were performed by live imaging at 37 °C using the LSM 710 (63x/1.46 Oil Plan-Apochromatic objective). Experimental cells or regions of interest (ROI) were chosen based on cell confluence, cell-cell contact (allowing gap junction communication) and comparable fluorescence intensity of neighbouring cells. Selected cells were photo-bleached using the 408 nm laser at 10% laser power. Fifteen iterations at 10-second intervals achieved a 50% decrease of ROI fluorescence intensity. Fluorescence recovery was assessed under the 488 nm laser. Images were captured at 10-second intervals and fluorescence intensity of the ROI was quantified over time and normalized to that of control, unbleached, calcein-loaded cells.

### Statistical analysis

Data were reported as mean ± standard deviation and statistical analyses, using Student’s t-test, were performed using Microsoft Excel of the Microsoft Office Suite. A *p*-value of less than 0.05 was considered significant.

## Electronic supplementary material

Supplementary Figure S1
