# Supplementary material for: Electronic Cigarette Smoke Impairs Normal Mesenchymal Stem Cell Differentiation
Source: Sci Rep. 2017 Oct 27;7:14281. doi: 10.1038/s41598-017-14634-z (PMC5660168; doi:10.1038/s41598-017-14634-z)

Electronic Cigarette Smoke Impairs Normal Mesenchymal Stem Cell Differentiation.

A. Shaito, J. Saliba, A. Husari, M. El-Harakeh, H. Chhouri, Y. Hashem, A. Shihadeh and M. El-Sabban.

Supplemnetary Figure S1.

**Supplementary Figure S1.**
**Original Full Length Blots.**

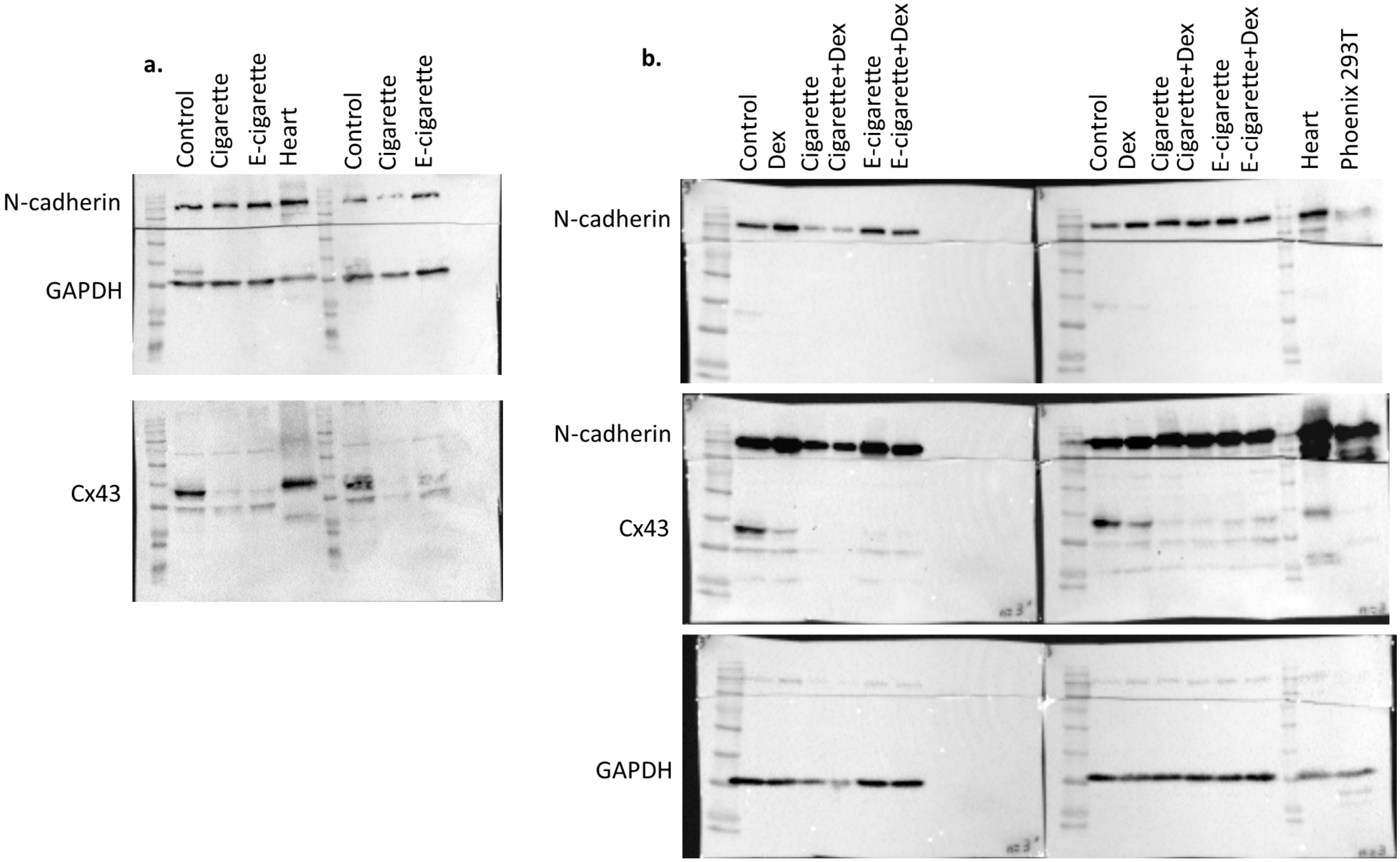

Supplement: Supplementary file 1 — Supplementary Figure S1 [file 41598_2017_14634_MOESM1_ESM.pdf]
